# Phase I study of the gamma secretase inhibitor PF-03084014 in combination with docetaxel in patients with advanced triple-negative breast cancer

**DOI:** 10.18632/oncotarget.13727

**Published:** 2016-11-30

**Authors:** Marzia A. Locatelli, Philippe Aftimos, E. Claire Dees, Patricia M. LoRusso, Mark D. Pegram, Ahmad Awada, Bo Huang, Rossano Cesari, Yuqiu Jiang, M. Naveed Shaik, Kenneth A. Kern, Giuseppe Curigliano

**Affiliations:** ^1^ Division of Experimental Therapeutics, European Institute of Oncology, Milan, Italy; ^2^ Medical Oncology Clinic, Institut Jules Bordet, Université Libre de Bruxelles, Brussels, Belgium; ^3^ Department of Hematology and Oncology, University of North Carolina, Lineberger Comprehensive Cancer Center, Chapel Hill, NC, USA; ^4^ Medical Oncology, Karmanos Cancer Institute, Detroit, MI, USA; ^5^ Breast Cancer Research Program, Stanford Cancer Institute, Stanford, CA, USA; ^6^ Pfizer Oncology, Groton, CT, USA; ^7^ Pfizer Oncology, Milan, Italy; ^8^ Pfizer Oncology, San Diego, CA, USA; ^9^ Yale Cancer Center, New Haven, CT, USA

**Keywords:** breast cancer, triple-negative, PF-03084014, gamma secretase, NOTCH signaling

## Abstract

**Background:**

The NOTCH signaling pathway may be involved in the survival of stem cell-like tumor-initiating cells and contribute to tumor growth. In this phase Ib, open-label, multicenter study (NCT01876251), we evaluated PF-03084014, a selective gamma-secretase inhibitor in patients with advanced triple-negative breast cancer.

**Methods:**

The dose-finding part was based on a 2×3 matrix design using the modified toxicity probability interval method. Oral PF-03084014 was administered twice daily continuously in combination with intravenous docetaxel given on day 1 of each 21-day cycle. Primary endpoint was first-cycle dose-limiting toxicity (DLT) for the dose-finding part and 6-month progression-free survival (PFS) for the expansion cohort treated at the maximum tolerated dose (MTD). Secondary endpoints included safety, objective response, and pharmacokinetics of the combination.

**Results and Conclusions:**

The MTD was estimated to be PF-03084014 100 mg twice daily / docetaxel 75 mg/m2. At this dose level, combination treatment was generally well tolerated (one DLT, grade 3 diarrhea, among eight DLT-evaluable patients). The most common all-grade, treatment-related adverse events reported in all patients (N = 29) were neutropenia (90%), fatigue (79%), nausea (72%), leukopenia (69%), diarrhea (59%), alopecia (55%), anemia (55%), and vomiting (48%). No effect was observed on the pharmacokinetics of docetaxel when administered in combination with PF-03084014. Four (16%) of 25 response-evaluable patients achieved a confirmed partial response; nine (36%) patients had stable disease, including five patients with unconfirmed partial response. In the expansion cohort, median PFS was 4.1 (95% CI 1.3-8.1) months (6-month PFS rate 17.1% [95% CI 0.8-52.6%]).

## INTRODUCTION

Triple-negative breast cancer (TNBC) is defined by the lack of estrogen (ER), progesterone (PR), and human epidermal growth factor 2 (HER2) receptors, and it accounts for approximately 15% of all breast cancer cases. Metastatic TNBC (mTNBC) is associated with a poor prognosis [1–3]. TNBCs represent a heterogeneous group of tumors with one common clinical feature: a distinctly aggressive nature with higher rates of relapse and shorter overall survival in the metastatic setting compared with other breast cancer subtypes. Because of the absence of well-defined molecular targets, cytotoxic chemotherapy is currently the standard treatment option for TNBC. Progression on first-line taxane therapy often occurs relatively early in mTNBC, with a median progression-free survival (PFS) of about 5-6 months [1–3]. Gene expression profiling identified six TNBC subtypes displaying unique gene expression and ontologies, including two basal-like, an immunomodulatory, a mesenchymal, a mesenchymal stem-like, and a luminal androgen receptor subtype [4].

It has been hypothesized that the poor response to standard treatment seen in patients with TNBC may be related to the presence of stem cell-like tumor-initiating cells refractory to cytotoxic chemotherapy [3, 5, 6]. The NOTCH signaling pathway is involved in the survival of stem cell-like tumor-initiating cells, as it plays an important role in normal stem cell development and differentiation, tumor growth regulation, and malignant cell survival [7–11]. Upregulation of the NOTCH pathway occurs in mesenchymal and mesenchymal stem-like TNBCs. Further, high levels of NOTCH and JAG1 were detected in tumors with poor-prognosis features and found associated with shorter survival compared to breast cancer patients with low tumor levels of NOTCH1 and JAG1 [7–9].

PF-03084014 is a reversible, noncompetitive, selective gamma-secretase inhibitor that blocks the NOTCH signaling pathway. It has demonstrated antitumor activity in preclinical xenografts models and in patients with solid tumors, including advanced thyroid cancer and desmoid tumors, and T-cell acute lymphoblastic leukemia [12–15]. Combination of PF-03084014 with docetaxel significantly increased inhibition of tumor growth in patient-derived and cell-line xenograft models of TNBC [13], prompting evaluation of this combination for the treatment of patients with TNBC.

This phase I study (A8641016) was designed to estimate the maximum tolerated dose (MTD) of PF-03084014 in combination with docetaxel, and evaluate safety, tolerability, pharmacokinetics, and antitumor activity in patients with advanced TNBC.

## RESULTS

### Patients

Twenty-two patients with mTNBC or hormone-refractory ER/PR-receptor positive breast cancer (two ER+ and one PR+), who had received up to two lines of prior therapy in the metastatic setting, were treated during dose escalation at three dose levels: eight at dose 2b (PF-03084014 100 mg twice daily [BID]/docetaxel 75 mg/m2), three at dose 3a (PF-03084014 100 mg BID/docetaxel 100 mg/m2), and 11 at dose 3b (PF-03084014 150 mg BID/docetaxel 75 mg/m2) (Supplemental Table S1 and Table S2). Seven additional patients with mTNBC were treated in first-line at dose 2b (estimated MTD) in the expansion phase. All treated patients (N = 29) were evaluable for safety, and 25 were evaluable for objective response.

Patient baseline demographic and disease characteristics are presented in Table 1. The majority of patients had ECOG performance status 0 (62%) and metastatic disease (86%), and received combination study treatment as first-line therapy (69%) in the advanced/metastatic setting.

**Table 1:** Patient baseline characteristics

Characteristic	PF-03084014 100 mg BID/ docetaxel 75 mg/m^2^ (*n* = 15)	PF-03084014 100 mg BID/ docetaxel 100 mg/m^2^ (*n* = 3)	PF-03084014 150 mg BID/ docetaxel 75 mg/m^2^ (*n* = 11)	All dose levels (N = 29)
Mean (range) age, years	53 (34-76)	43 (32-64)	46 (27-69)	50 (27-76)
Race, n (%) White Black	14 (93) 1 (7)	3 (100) 0	11 (100) 0	28 (97) 1 (3)
ECOG PS, n (%) 0 1	9 (60) 6 (40)	1 (33) 2 (67)	8 (73) 3 (27)	18 (62) 11 (38)
Disease, n (%) Metastatic Locally recurrent	14 (93) 1 (7)	3 (100) 0	8 (73) 3 (27)	25 (86) 4 (14)
Prior cancer surgery, n (%)	15 (100)	3 (100)	11 (100)	29 (100)
Prior systemic treatmenta, n (%) No Yes	10 (67) 5 (33)	3 (100) 0	7 (64) 4 (36)	20 (69) 9 (31)
Prior radiation therapya, n (%) No Yes	11 (73) 4 (27)	3 (100) 0	9 (82) 2 (18)	23 (79) 6 (21)
Prior taxane therapy, n (%) Yes (adjuvant) Yes (neoadjuvant) Yes (advanced)	6 (40) 4 (27) 0	1 (33) 0 0	4 (36) 1 (9) 0	11 (38) 5 (17) 0

ECOG PS, Eastern Cooperative Oncology Group performance status.

^a^Prior anticancer treatment in the advanced/metastatic setting.

### Dose-limiting toxicity (DLT) and safety profile

Patients received treatment according to the study design [16] (Supplemental Information). Of 22 DLT-evaluable patients, one (13%) patient at dose 2b, one (33%) patient at dose 3a, and four (36%) patients at dose 3b experienced DLTs, including grade 3 diarrhea (dose 2b); grade 3 diarrhea (dose 3a); and grade 3 dehydration, grade 3 nausea, grade 4 febrile neutropenia, and grade 5 septic shock (dose 3b) (Supplemental Table S2). All grade 3-4 DLTs resolved following dose reduction of PF-03084014 and docetaxel. Only three patients were treated at dose 3a due to concerns related to potential toxicity of treatment with docetaxel 100 mg. The patient, who died of septic shock at dose 3b, had developed grade 4 neutropenia 7 days after starting study combination treatment, indicating an event potentially related to docetaxel-induced bone marrow toxicity. A second, non-treatment-related death was reported at dose 2b in a 67-year-old patient who experienced respiratory distress and fatal disease progression. The MTD for the combination, defined as the highest tested dose level with a DLT rate < 0.33, was estimated to be PF-03084014 100 mg BID/docetaxel 75 mg/m2 based on the DLTs observed at each dose level.

The most common all-grade, treatment-related adverse events (AEs) for the combination of PF-03084014 with docetaxel reported in all patients (N = 29) were neutropenia (90%), fatigue (79%), nausea (72%), leukopenia (69%), diarrhea (59%), alopecia (55%), anemia (55%), and vomiting (48%) (Table 2). PF-03084014/docetaxel combination treatment-related grade 3-4 neutropenia was observed in 25 (86%) patients, grade 3-4 leukopenia in 19 (66%) patients, and grade 3-4 febrile neutropenia in eight (28%) patients; no treatment-related grade ≥ 3 thrombocytopenia was reported. Other grade 3-4 combination treatment-related AEs noted included fatigue, nausea, diarrhea, anemia, vomiting, mucosal inflammation, and hypophosphatemia (Table 2). At the MTD, the most frequently reported non-hematologic grade 3-4 laboratory abnormalities were hypophosphatemia (33%) and hypokalemia (13%).

**Table 2:** Treatment-related adverse events occurring in more than 20% of patients (N = 29)a

Adverse event	Grade 1 *n* (%)	Grade 2 *n* (%)	Grade 3b *n* (%)	Grade 4 *n* (%)	All grades *n* (%)
Neutropeniac	0	1 (3)	1 (3)	24 (83)	26 (90)
Fatigue	6 (21)	15 (52)	2 (7)	0	23 (79)
Nausea	12 (41)	7 (24)	2 (7)	0	21 (72)
Leukopeniad	0	1 (3)	13 (45)	6 (21)	20 (69)
Diarrhea	5 (17)	9 (31)	3 (10)	0	17 (59)
Alopecia	9 (31)	7 (24)	0	0	16 (55)
Anemia	7 (24)	8 (28)	1 (3)	0	16 (55)
Vomiting	9 (31)	4 (14)	1 (3)	0	14 (48)
Mucosal inflammation	8 (28)	4 (14)	1 (3)	0	13 (45)
Rashe	8 (28)	5 (17.2)	0	0	13 (45)
Hypophosphatemia	3 (10)	4 (14)	4 (14)	0	11 (38)
Febrile neutropenia	0	0	2 (7)	6 (21)	8 (28)
Thrombocytopeniaf	5 (17)	4 (14)	0	0	9 (31)
Pyrexia	7 (24)	0	0	0	7 (24)
Stomatitis	3 (10)	4 (14)	0	0	7 (24)
Constipation	5 (17)	1 (3)	0	0	6 (21)
Decreased appetite	2 (7)	4 (14)	0	0	6 (21)
Headache	5 (17)	1 (3)	0	0	6 (21)
Proteinuria	2 (7)	4 (14)	0	0	6 (21)

^a^‘Treatment-related’ indicates that the adverse event has been related to PF-03084014 and/or docetaxel.

^b^Three patients developed grade 3 pneumonia, and one patient each experienced aspartate aminotransferase elevation, dehydration, hyponatremia, colitis, hypokalemia, prolonged prothrombin time, and decreased performance status (all grade 3). One grade 5 treatment-related adverse event (septic shock) was reported in a patient who received PF-03084014 150 mg BID/docetaxel 75 mg/m2.

^c^Includes neutropenia and decreased neutrophil count.

^d^Includes leukopenia and decreased white blood cell count.

^e^Includes rash, erythematous rash, maculopapular rash, acne, and dermatitis acneiform.

^f^Includes thrombocytopenia and decreased platelet count.

The majority (72%) of patients discontinued PF-03084014 due to disease progression. Four patients permanently discontinued PF-03084014 owing to a treatment-related AE: one patient (dose 3a) due to grade 3 vomiting and three patients (dose 3b) due to grade 2 fatigue, grade 3 pneumonia, and grade 4 febrile neutropenia (n = 1 each). Overall, median duration of treatment was four (range, 1-12) cycles for all patients and four (range, 2-12) cycles at the MTD.

### Pharmacokinetics

**Figure 1:**
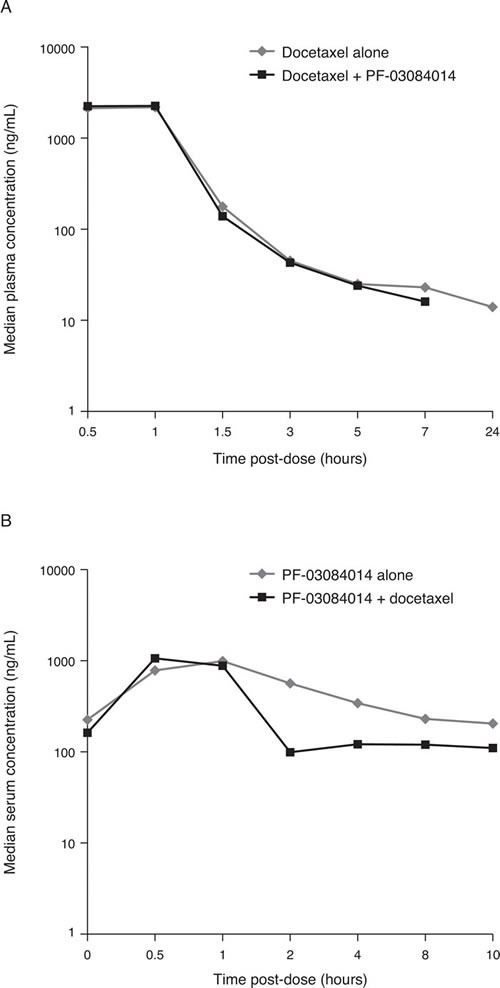
Median concentration-time profiles for docetaxel A. and PF-03084014 B.

No effect was observed on the pharmacokinetics of docetaxel when administered in combination with PF-03084014 (Figure 1A). Median serum concentration-time profiles of PF-03084014 alone or in combination with docetaxel, at the MTD, are shown in Figure 1B. A lower, transient exposure to PF-03084014 was observed on days in which it was dosed in combination with docetaxel. At the MTD, maximum mean observed serum concentration of PF-03084014 was 963 ng/ml when administered alone and 950 ng/ml in combination with docetaxel.

### Antitumor activity

**Figure 2:**
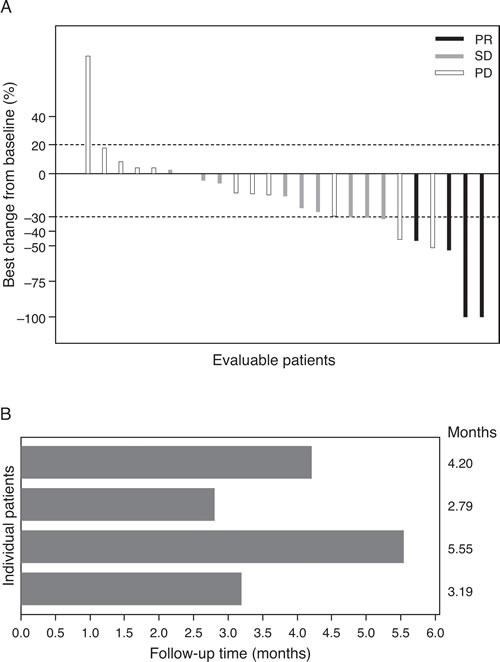
Best tumor size change from baseline A. Duration of response to treatment with PF-03084014 and docetaxel B. PR, partial response; PD, progressive disease; SD, stable.

Best change in target lesions from baseline for each response-evaluable patient is shown in Figure 2A. Four (16%; 95% CI 4.5-36.1%) of 25 response-evaluable patients achieved a confirmed partial response, with response duration as shown in Figure 2B. Two of these patients received the combination at the MTD, and one had been previously treated with paclitaxel in the adjuvant setting. Nine (36%) patients had stable disease, including five patients with unconfirmed partial response. Eleven (44%) patients had best overall response of progressive disease (Supplementary Table S3). In the expansion cohort, median PFS was 4.1 (95% CI 1.3-8.1) months, with a 6-month PFS rate of 17.1% (95% CI 0.8-52.6%).

## DISCUSSION

We report here the results from the first phase I trial of the novel gamma-secretase inhibitor PF-03084014 in combination with docetaxel in patients with locally advanced or metastatic TNBC. Results from preclinical studies had demonstrated antitumor activity of this combination in xenograft models of TNBC [13].

Following dose escalation/de-escalation of either drug, the MTD for the combination in patients with TNBC was estimated to be PF-03084014 100 mg BID plus docetaxel 75 mg/m2. At the estimated MTD, combination treatment was generally well tolerated: one DLT, grade 3 diarrhea, was noted in one of the eight DLT-evaluable patients treated at this dose level. At higher doses, a patient in the PF-03084014 150 mg BID/docetaxel 75 mg/m2 cohort C experienced grade 5 treatment-related septic shock, potentially related to docetaxel-induced neutropenia. The plasma exposure of docetaxel for this patient was greater than the mean exposure of the group. No unexpected toxicities were observed compared to results of prior studies conducted with single-agent PF-03084014 in patients with solid tumors or hematologic malignancies [14, 15] or with docetaxel [1]. The most frequent treatment-related AEs reported for the combination of PF-03084014 and docetaxel across dose levels were neutropenia, fatigue, nausea, leukopenia, diarrhea, alopecia, anemia, and vomiting. The non-hematologic events were generally mild to moderate in severity with only grade 3 diarrhea reported frequently. Treatment with PF-03084014 in combination with docetaxel was not limited by severe diarrhea, as opposed to what was observed with other drugs from the same class, MK-0752 and RO4929097, administered as single agents [17, 18]. In addition, development of squamous cell skin cancers was not observed after treatment with PF-03084014 either in combination with docetaxel or as a single agent [14].

No effect was observed in this study on the pharmacokinetics of docetaxel when administered in combination with PF-03084014. A transient decrease in exposure was noted for PF-03084014 during the days in which it was administered in combination with docetaxel. Prophylactic administration of dexamethasone from day -1 to 2 of each treatment cycle may potentially explain this lower exposure observed for PF-03084014 on the combination dosing day, resulting from an effect on metabolism and drug transport processes [19–21], but no clinical impact is foreseen because of continuous PF-03084014 administration.

Four (16%) patients had a confirmed partial response in first-line treatment of advanced disease; nine (36%) patients achieved stable disease as best response, including five patients with unconfirmed partial response. The overall response rate observed in this study appears low, potentially reflecting the limited number of patients treated in first-line with the combination at the MTD. In addition, three of the patients treated in first-line at the estimated MTD had a dose reduction of PF-03084014 and/or docetaxel. Previous results of phase I studies conducted with the gamma-secretase inhibitor RO4929097 in combination with gemcitabine or capecitabine showed limited antitumor activity in patients with advanced solid malignancies [22, 23]. One (6%) patient with nasopharyngeal cancer had a partial response following treatment with the RO4929097/gemcitabine combination at a dose above the recommended phase II dose [22]. Three (10%) patients with colon or cervical cancer had a partial response to the RO4929097/capecitabine combination [23].

## CONCLUSIONS

Treatment of patients with locally advanced or metastatic TNBC with the gamma-secretase inhibitor PF-03084014 in combination with docetaxel demonstrated a manageable safety profile and limited preliminary antitumor activity. The MTD for the combination was estimated to be PF-03084014 100 mg BID/docetaxel 75 mg/m2.

## PATIENTS AND METHODS

### Study design and patients

This phase Ib, open-label, multicenter study was conducted in patients with advanced TNBC and divided into two parts: (1) a dose-finding component in patients who had received up to two prior lines of chemotherapy for metastatic disease, and (2) an expansion cohort restricted to patients treated in the first-line, advanced setting at the estimated MTD.

Adult women with advanced/metastatic TNBC were eligible for the study if they had documented ER-negative, PR-negative, and HER2-negative status (dose-escalation and expansion cohort) or were ER/PR-positive, HER2-negative, and were unresponsive to hormonal therapy by investigator assessment (dose-escalation). Further, eligible patients were required to have at least one measurable lesion by RECIST version 1.1 criteria, Eastern Cooperative Oncology Group (ECOG) performance status 0 or 1; and adequate renal, liver, and bone marrow function.

Patients were excluded if they had symptomatic brain metastases requiring steroids, had received systemic anticancer treatment within 3 weeks of study entry (6 months for taxanes), had been previously treated with a gamma-secretase inhibitor or NOTCH signaling inhibitor, had a corrected QT (QTc) interval > 470 msec, or had current use or anticipated need for treatment with moderate/strong cytochrome P450 (CYP) 3A4 inhibitors or strong CYP3A4 inducers. Further, patients who were candidates for resection or radiation therapy with curative intent were not eligible.

Approval was obtained from the ethics committees of the participating institutions. Patients gave written informed consent. The study (NCT01876251) followed the Declaration of Helsinki and the International Conference on Harmonization Good Clinical Practice guidelines.

The primary endpoint was first-cycle DLT attributable to PF-03084014 in combination with docetaxel for the dose-finding part of the study and PFS at 6 months for the expansion cohort. Hematologic DLT included grade 4 neutropenia lasting > 7 days, febrile neutropenia, grade ≥ 3 neutropenic infection, grade ≥ 3 thrombocytopenia with bleeding, and grade 4 thrombocytopenia without bleeding, if attributable to PF-03084014 in combination with docetaxel. Non-hematologic DLT included grade ≥ 3 AEs if maximally treated; grade 3 QTc prolongation ( > 500 msec) persisting after correction of any reversible causes; dose delay of 2 weeks due to treatment-related AEs; and failure to deliver at least 80% of the planned dose in first cycle due to treatment-related AEs, if attributable to PF-03084014 in combination with docetaxel. Secondary study endpoints were safety, objective response, duration of response, and pharmacokinetics of PF-03084014 in combination with docetaxel.

### Study treatment

PF-03084014 was administered orally twice daily (BID) continuously in combination with docetaxel given intravenously on day 1 of each 21-day cycle. Steroid premedication was given from day -1 to day 2 of each cycle, as required for docetaxel administration. Administration of PF-03084014 in the dose-finding component started on day 2 of cycle 1 for pharmacokinetics evaluation purposes. In the expansion cohort, patients received PF-03084014 and docetaxel at the estimated MTD starting on day 1 of each 21-day cycle, with no specific timing requirement for the administration of PF-03084014 relative to docetaxel. Study treatment was given until disease progression, patient withdrawal, or unacceptable toxicity. Treatment with granulocyte-colony stimulating factor (G-CSF) was allowed in patients with neutropenia. Prophylactic use of G-CSF was permitted only after cycle 1.

### Study assessments

AEs were continuously monitored and graded for severity using the National Cancer Institute Common Terminology Criteria for Adverse Events (CTCAE) version 3.0.

Antitumor activity was assessed by computed tomography or magnetic resonance imaging and bone scans at baseline, every 6 weeks, until disease progression or death, patient refusal, start of another anticancer treatment, or until 1 year from cycle 1, day 1, of last enrolled patient whichever occurred first, using RECIST version 1.1.

Blood samples to determine the pharmacokinetics of PF-03084014 were collected on days 2, 8, and 21 (days 1 and 21 in the expansion cohort) of cycle 1, and on day 1 of subsequent cycles. In the dose-finding component, blood samples were collected on day 1 of cycles 1 and 2 to evaluate the pharmacokinetics of docetaxel. Samples were analyzed for PF-03084014 and docetaxel concentrations using validated analytical methods.

### Statistical methods

Dose escalation/de-escalation followed a 2×3 matrix design with the modified toxicity probability interval method [16] (mTPI, see Supplemental Methods) using doses of PF-03084014 and docetaxel shown in Supplemental Table S1. The estimated MTD was defined as the highest tested dose level with estimated DLT rate < 0.33.

The objective response rate was summarized with exact two-sided 95% confidence interval (CI) calculated using a method based on the F distribution. PFS was summarized by the Kaplan-Meier method with 95% CI for the median calculated using the Brookmeier-Crowley method.

European Clinical Trial Database (EudraCT) Number: 2013-000659-41

## SUPPLEMENTARY MATERIALS FIGURES AND TABLES


