# Supplementary material for: Phase I study of the gamma secretase inhibitor PF-03084014 in combination with docetaxel in patients with advanced triple-negative breast cancer
Source: Oncotarget. 2016 Nov 30;8(2):2320–8. doi: 10.18632/oncotarget.13727 (PMC5356802; doi:10.18632/oncotarget.13727)
Supplement: Supplementary file 1 [file oncotarget-08-2320-s001.pdf]

Phase I study of the gamma secretase inhibitor PF-03084014 in combination with docetaxel in patients with advanced triple-negative breast cancer

Supplementary Material

Supplemental Methods: Study design and analysis

Supplemental Table S1: Potential dose combinations

Supplemental Table S2: Dose-limiting toxicities in first treatment cycle

Supplemental Table S3: Response to treatment with PF-03084014 and docetaxel by dose level (n = 25)

Supplemental Methods

Study Design

A modified toxicity probability interval (mTPI) study design based on a Bayesian statistics framework and a beta/binomial hierarchical model was used to determine dose escalation/de-escalation in the dose-finding part of this study. If the toxicity rate at the selected dose level was far smaller than the target rate (pT = 0.30), the mTPI would recommend escalating the dose level; if it was close to pT, the mTPI would recommend continuing at the selected dose; if it was far greater than pT, the mTPI would recommend deescalating the dose level.

Reference: Ji Y et al. A modified toxicity probability interval method for dose-finding trials. *Clin Trials.* 2010; 7: 653–663.

Analysis

The safety analysis set included all enrolled patients who received at least one dose of study medication. Patients who were lost to follow-up before receiving at least 80% of

the planned first-cycle dose due to reasons unrelated to treatment were not evaluable for DLT. The response analysis set included all enrolled patients who had baseline assessments and at least one on-study tumor assessment. Patients who were treated and removed from the study prior to on-study tumor assessment because of disease progression were considered evaluable for efficacy and counted as treatment failures.

Supplemental Table S1: Potential dose combinations

Dose level	PF-03084014 (mg BID)	Docetaxel (mg/m^2)
1	80	75
2a	80	100
2b	100 (starting dose)	75 (starting dose)
3a	100	100
3b	150	75
4	150	100

BID, twice daily.

Supplemental Table S2: DLTs in first treatment cycle

	**PF-03084014 100 mg BID/docetaxel 75 mg/m^2 (*n* = 8)**	**PF-03084014 100 mg BID/docetaxel 100 mg/m^2 (*n* = 3)**	**PF-03084014 150 mg BID/docetaxel 75 mg/m^2 (*n* = 11)**
DLT-evaluable patients, *n*	8	3	11
DLT events, *n* (%)	1 (13%)<br>Grade 3 diarrhea	1 (33%)<br>Grade 3 diarrhea and grade 4 febrile neutropenia	4 (36%)<br>Grade 3 dehydration, grade 3 nausea, grade 4 febrile neutropenia, grade 5 septic shock

DLT, dose-limiting toxicity.

Supplemental Table S3: Best overall response to treatment with PF-03084014 and docetaxel by dose level (*n* = 25, response-evaluable set)

	**PF-03084014 100 mg BID/ docetaxel 75 mg/m^2 (*n* = 14)**	**PF-03084014 100 mg BID/ docetaxel 100 mg/m^2 (*n* = 3)**	**PF-03084014 150 mg BID/ docetaxel 75 mg/m^2 (*n* = 8)**	**All dose levels (*N* = 25)**
Complete response, *n* (%)	0	0	0	0
Partial response, *n* (%)	2 (14)	0	2 (25)	4 (16)
Stable disease, *n* (%)	6 (43)	1 (33)	2 (25)	9 (36)
Progressive disease, *n* (%)	6 (43)	2 (67)	3 (38)	11 (44)
Indeterminate[a], *n* (%)	0	0	1 (13)	1 (4)
Objective response rate, *n* (%)<br>95% exact CI	2 (14)<br>1.8–42.8	0<br>0.0–70.8	2 (25)<br>3.2–65.1	4 (16)<br>4.5–36.1

[a]Indeterminate response due to stable disease prior to new anticancer treatment on study day 37, thus earlier than 6 weeks. CI, confidence interval.
